# Mitoquinone shifts energy metabolism to reduce ROS-induced oxeiptosis in female granulosa cells and mouse oocytes

**DOI:** 10.18632/aging.204475

**Published:** 2023-01-09

**Authors:** Kuan-Hao Tsui, Chia-Jung Li

**Affiliations:** 1 Department of Obstetrics and Gynaecology, Kaohsiung Veterans General Hospital, Kaohsiung 813, Taiwan; 2 Institute of Biopharmaceutical Sciences, National Sun Yat-Sen University, Kaohsiung 804, Taiwan; 3 Department of Obstetrics and Gynaecology, National Yang-Ming University School of Medicine, Taipei 112, Taiwan; 4 Department of Obstetrics and Gynecology, Taipei Veterans General Hospital, Taipei 112, Taiwan; 5 Department of Pharmacy and Master Program, College of Pharmacy and Health Care, Tajen University, Pingtung County 907, Taiwan; 6 Department of Medicine, Tri-Service General Hospital, National Defense Medical Center, Taipei 114, Taiwan; 7 College of Health and Nursing, Meiho University, Pingtung County 912, Taiwan

**Keywords:** MitoQ, oxeiptosis, ROS, mitochondria, metabolism

## Abstract

The female reproductive system is quite sensitive to regulation, and external environmental stimuli may cause oxidative stress which in turn may lead to accelerated aging and programmed cell death in female reproductive cells. The aim of this study was to investigate whether or not mitoquinone (MitoQ) could resist ROS-induced apoptosis in human granulosa cells and mouse oocytes. We found that the MitoQ treatment significantly reduced production of reactive oxygen species (ROS) and imbalance in mitochondrial membrane potential. The MitoQ treatment prevented an excessive mitochondrial fragmentation by upregulating Drp1 S637 and decreasing Drp1 S637 phosphorylation. More importantly, MitoQ maintained aerobic respiration and reduced anaerobic respiration by regulating reprogramming of intracellular energy metabolism, which enhanced cellular ATP production. MitoQ effectively reduced the expressions of AIFM1 and PGAM5, key molecules whose expressions were reversed not only in granulosa cells but also in mouse oocytes. Our findings suggest that MitoQ can ameliorate the mitochondrial deterioration caused by ROS and reprogram cellular energy metabolism, providing protection to cells against apoptosis. The presence of MitoQ may help in protecting human germ cells under *in vitro* culture conditions.

## INTRODUCTION

Reactive oxygen species (ROS) are a byproduct of cellular metabolism and play an important role in follicular and embryonic development [1, 2]. ROS in follicular fluid are mainly produced by ovarian granulosa cells, macrophages, and follicular membrane cells, while excess free radicals can be produced by impaired oocyte metabolism. Therefore, the ROS level in follicular fluid can reflect the metabolic status of follicles [3, 4]. However, exposure to highly polluted and pathogenic environments causes an increased protein fold load, fatty acid oxidation, and an imbalance in energy metabolism, thus accumulating ROS in endoplasmic reticulum, peroxisomes, and mitochondria, which cannot be removed by antioxidant enzymes [5]. This irreversibly changes protein and DNA mutations, resulting in the loss of cellular function. Previous studies have shown that ROS can cause apoptosis, necroptosis, activation of inflammasome pathways, and caspase-dependent cell deaths, while different cells may exhibit different tolerance to ROS [6–8].

Oxeiptosis is a novel oxygen-free, radical-induced, caspase-independent programmed cell death. The key molecule regulated was the activation of the KEAP1/PGAM5/AIFM1 pathway to drive cell death. Once the intracellular ROS sensor Keap1 receives the message, the cytoprotective mechanism is activated, and the Keap1/NRF2/PGAM5 complex is dissociated. In cases of severe cell damages, PGAM5 dephosphorylates AIFM1, which inactivates intracellular caspases. PGAM5 has the activity of RIPK proteins that regulates necroptosis and induces a variety of programmed cell death pathways [7, 9–12]. The molecular mechanism underlying this novel oxeiptosis in human pathophysiology remains largely unknown.

Antioxidants are currently one of the most effective strategies for maintaining intracellular redox homeostasis and improving the physiological functions of germ cells [13, 14]. Since most antioxidants have difficulty getting into the body to work, higher doses are required to achieve antioxidant effects. The mitochondria-targeted antioxidant mitoquinone (MitoQ), a ubiquinone derivative, has similar results to CoQ10, except that MitoQ is a lipophilic triphenylphosphonium moiety (TPP+) cation, which allows MitoQ to more easily cross the mitochondrial membrane and accumulate in the mitochondria, and is also available for mitochondrial respiratory chain complex II. In an environment where MitoQ accumulates stably in the mitochondria, it has an antioxidant effect under the action of continuous reduction [15]. MitoQ has been reported to promote maturation of porcine oocytes *in vitro* by maintaining mitochondrial homeostasis and suppressing UCP2 levels to maintain the stability of mitochondrial heat production [16, 17]. In addition, MitoQ has been reported to reduce ROS produced by vitrification and freezing of oocytes, thereby increasing cell viability and fertility [18]. In addition to protecting the mitochondria, MitoQ may improve spindle and chromosome defects in oocytes exposed to oxidative stress and in oocytes from aged mice during *in vitro* maturation (IVM) [19].

In this study, we used HGL5 cells and mouse oocytes in order to investigate the protective effects of MitoQ on ROS-induced apoptosis. We also quantified the effects of MitoQ on energy production, lactate production, and glucose metabolism and analyzed the relationship between glycolysis and oxidative phosphorylation conversion. Our results contribute to the theoretical basis of the novel and non-canonical cell death-executing signaling pathways provided by MitoQ in germ cells.

## MATERIALS AND METHODS

### Cell culture and treatment protocol

The human ovarian granulosa cell line (HGL5) was purchased from Applied Biological Materials Inc. HGL5 cells were maintained in an incubator containing 10% FBS, 1% penicillin/streptomycin, 2% Ultroser G (Pall Corp.), and 1% ITS Plus (Zen-Bio). HGL5 cells were stabilized in a humidified incubator with 5% CO_2_ maintained at 37°C. In the present study, we used H_2_O_2_ to induce apoptosis. The cells were pretreated with or without 10 nM MitoQ for 20 h, followed by 0.8 mM H_2_O_2_ for 4 h, and the protective effect of MitoQ on germ cells was analyzed.

### Mitochondrial function measurement

Mitochondrial function assays were performed as previously described [20]. Cells from each group were collected and stained with DCFDA (5 μM), MitoTracker green (10 nM), tetramethylrhodamine methyl ester (TMRM; 200 nM), MitoSOX (5 μM) (Molecular Probes), and ATP (BioTracker ATP- Red Live Cell Dye, Merck) at 37°C in an incubator. The cells were washed with PBS to remove excess fluorescent dye, and the centrifuged cell pellets were re-suspended in PBS for flow cytometry analysis.

### Cell death detection by Annexin V-FITC/PI double staining

The oxygen consumption rate was determined as previously described [20]. Briefly, cells were collected, washed twice with PBS, re-suspended in binding buffer, and stained with Annexin V-FITC and PI (GeneTex; GTX85591) solution for 15 min at room temperature in the dark. After incubation at 37°C, 1 mL of binding buffer was added, and cells were analyzed using a flow cytometry (FACSCalibur, BD Bioscience, CA, USA).

### Oxygen consumption rate measurement

The oxygen consumption rate was determined as previously described [21]. This was performed using an extracellular flux analyzer (Agilent Technologies, Santa Clara, CA, USA) and the Seahorse XF HS mini platform to measure oxygen consumption rate (OCR). Cells were seeded in trays and on average maintained at a density of 2000 cells per well. Changes in cellular respiration were assessed over time during the mitochondrial function assays, and oligomycin, FCCP, and antimycin A/rotenone were administered sequentially.

### Western blotting

Western blotting was performed as previously described [22]. The primary antibodies used were Drp-1 (Abcam; Ab56788), Drp-1 p-S616 (ABclonal; AP0849), Drp-1 p-S637 (Cell Signaling; 4867), AIFM1 (Arigo; ARG54387), AIFM1 p-S116 (ECM Biosciences; AP5501), NRF2 (Cell Signaling; 33649), KEAP1 (Proteintech; 10503-2-AP), and GAPDH (GeneTex; GTX627408).

### Time-lapse microscopy

To visualize cell morphological changes and organelle distribution, we used a 3D NanoLive microscope (3D Cell Explorer, NanoLive, Switzerland) for time-lapse photography. Human granulosa HGL5 cells were seeded at a density of 2 × 10^4^ cells/mL in glass-bottom culture dishes for clarity. Time-lapse photography was performed directly after the ROS treatment, and the cells were placed in a humidified chamber at 37°C and 5% CO_2_ for a continuous image capture.

### RNA extraction and real-time PCR

Samples were collected, and total RNA was extracted using the EasyPrep Total RNA Kit. Extracted RNA was reverse-transcribed to cDNA using the ToolScript MMLV RT Kit. Qualified RNA was reverse-transcribed using the TOOLS M-MLV RTase and ToolScript MMLV RT Kit with the StepOnePlus system (Applied Biosystems, Foster City, CA, USA). Nucleic acid reagents were purchased from TOOLS (Biotools, Taipei, Taiwan). All primer sequences are listed in Supplementary Table 1.

### Animal model and oocyte collection

Mice obtained from the National Laboratory Animal Center were used in this study. Aged C57BL/6J mice were housed in cages at 25°C on a 12-h light/12-h dark cycle with normal food and water intake. All animal studies were conducted in accordance with procedures approved by the Institutional Animal Care and Use Committee (#2021-2024-A050) of Kaohsiung Veterans General Hospital. For *in vivo* experiments, female mice (>40 weeks old) from C57BL/6J were superovulated by an injection of 5 IU equine chorionic gonadotrophin (eCG), followed by administration of 5 IU human chorionic gonadotropin (hCG) for 48 h. Approximately 14–16 h later, mice were anesthetized, oocytes were collected from the ovaries in the cumulus-oocyte complex, and oocytes were obtained by removing the cumulus mass in medium containing 0.5 mg/mL hyaluronidase. The oocytes were incubated at 37°C in a 5% CO_2_ incubator. The obtained oocytes were randomly divided equally into two groups: control and MitoQ (treated with 10 nM MitoQ for 24 h).

### Fluorescent staining of oocytes

Mouse oocytes were obtained by immersing the cumulus-oocyte complex in 500 μL of ICSI cumulase (Origio; No.1612) for 10 min. To test mitochondrial function in oocytes, oocytes were incubated in 10 μg/mL of JC-1 solution for 20 min at 37°C. The oocytes were then washed twice before a confocal microscope (Ex/Em = 585/590 nm) was used for imaging, whose intensity was analyzed using ImageJ software.

### UHPLC-MS/MS

To assess the effect of the metabolites, the procedure was performed as described in our previous publication [22]. All analytes were detected using electrospray ionization in positive ion multiple reaction-monitoring (MRM) mode. The quantitative and qualitative MRM transition ions were selected as the most abundant products and characteristic fragment ions, respectively.

### Statistical analysis

The data obtained in this study were subjected to at least three independent experiments, and all data are expressed in mean ± error values of repeated measurements. Semi-quantitative, mitochondrial, and 2.5D constructs were characterized and analyzed using ImageJ software (NIH), Zenlite software (Carl Zeiss Co. Ltd.), and MicroP software [6], respectively. Statistical significance was assessed using GraphPad Prism 8.0 (GraphPad Software, San Diego, CA, USA), followed by Tukey’s post hoc test to assess a significant difference between group means using a two-way analysis of variance test. Differences were considered statistically significant at *p* < 0.05.

## RESULTS

### MitoQ improves oxidative stress-induced mitochondrial dysfunction

**Figure 1:**
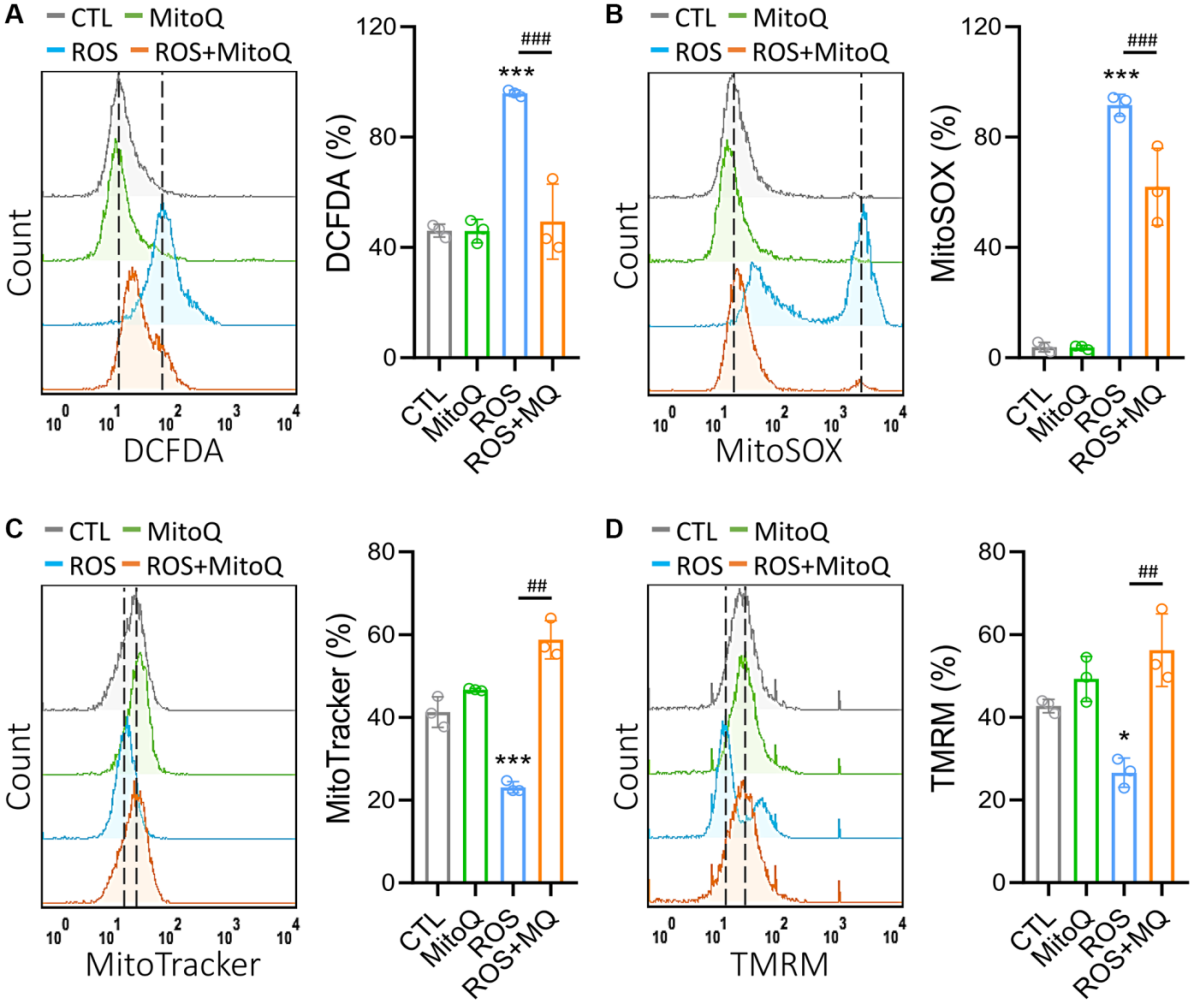
**MitoQ attenuates ROS-induced mitochondrial dysfunction in human granulosa cells.** We treated GC cells with MitoQ (10 nM) for 24 h and analyzed mitochondrial function. Measurements of (**A**) cellular, (**B**) mitochondrial ROS, (**C**) mitochondrial mass, and (**D**) mitochondrial membrane potential (Δψm) by flow cytometry. ^*^p < 0.05, and ^***^p < 0.001 compared to the control. ^##^*p* < 0.01 and ^###^*p* < 0.001 compared to ROS only.

To investigate the effects of MitoQ on intracellular H_2_O_2_ and mitochondrial O_2_^•−^, DCFH-DA and MitoSOX fluorescent dyes were detected (Figure 1A and 1B). These results indicated that MitoQ significantly decreased the intracellular and mitochondrial ROS levels in the cells. This in turn suggests that MitoQ protects the HGL5 cells from the oxidative stress damage by inhibiting the mitochondrial ROS production. We further described the effect of MitoQ on the mitochondrial mass and mitochondrial membrane potential (*Δψm*). Cells pretreated with MitoQ maintained ~60% of their mitochondrial mass compared to ~20% in the ROS group (Figure 1C). As was shown in Figure 1D, ROS significantly decreased TMRM fluorescence, which was indicative of *Δψm* loss and mitochondrial damage. After cells were pretreated with MitoQ, the HGL5 cells showed a significant increase in *Δψm*. These results suggest that MitoQ reduces the mitochondrial ROS levels and improves the mitochondrial quality and function.

### MitoQ ameliorates ROS-induced mitochondrial dynamic imbalance

**Figure 2:**
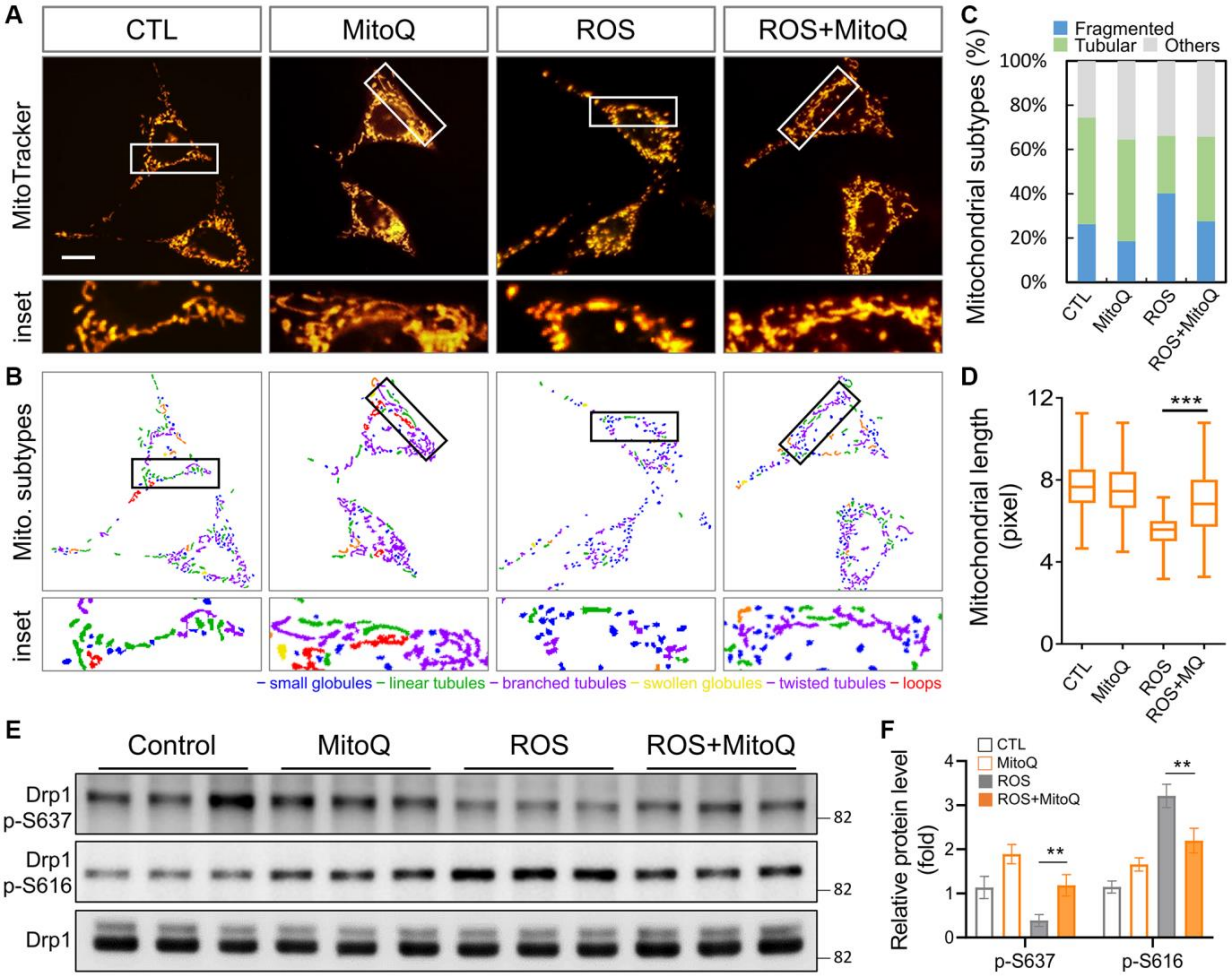
**Effects of MitoQ on imbalances in mitochondrial dynamics in ROS-exposed granulosa cells.** (**A**) Mitochondrial network was stained with MitoTracker Green and analyzed using a fluorescence microscopy with image acquisition. (**B**) The images below were divided into six major categories based on the morphology of the mitochondria. (**C**) Three main mitochondrial types were quantified: fragmented, tubular and others. (**D**) The total length of each mitochondrion was assessed. (**E**) The levels of mitochondrial fission protein Drp1 were analyzed by immunoblotting. (**F**) Quantification of Drp1 phosphorylated protein levels. ^**^p < 0.01 and ^***^p < 0.001.

Our results demonstrated that MitoQ effectively regulated the mitochondrial function (Figure 1). Therefore, we further analyzed the mitochondrial morphology (Figure 2A). The control groups stained with the mitochondrial stain MitoTracker showed elongated mitochondria. In contrast, mitochondrial fragmentation rose substantially after the H_2_O_2_ treatment, while mitochondrial fragmentation significantly improved with the MitoQ treatment. MicroP software was used to classify the mitochondria into the six categories of globules, tubules, branched, swollen, twisted, and loops. The differences were marked in different colors (Figure 2B). The magnified view clearly shows the distribution of mitochondria in each group with the different morphologies. The quantitative results in Figure 2C show that the number of fragmented mitochondria in the ROS group was significantly higher than those in the other three groups. Moreover, MitoQ was effective in reducing the fragmentation of mitochondria caused by ROS. The remaining four mitochondrial groups were classified as the other groups. As was shown in Figure 2D, ROS cause mitochondrial fragmentation, which is indicated by the splitting of organelles into spherical or short rod-like shapes. This effect is counteracted by the depletion of MitoQ, which limits the mitochondrial fragmentation caused by ROS. In addition, the average mitochondrial length was significantly greater in the MitoQ group than in the ROS group (Figure 2E). Phosphorylation of Drp1 at serine position 616 (S616) promotes mitochondrial fission, which facilitates its translocation to the mitochondria. Conversely, mitochondrial fission is inhibited by phosphorylation of S637. We analyzed the expression of the mitochondrial fission protein Drp1 using western blotting (Figure 2E). Following the quantification by western blotting, the amount of the S637-Drp1 phosphorylation fell in the ROS group, while the S637-Drp1 expression was higher in the ROS+MitoQ group than in the ROS group. However, the S616-Drp1 protein phosphorylation was extremely high in the ROS group, and the S616 expression significantly fell in the ROS+MitoQ group (Figure 2F).

### MitoQ protects cells from ROS-induced oxeiptosis

**Figure 3:**
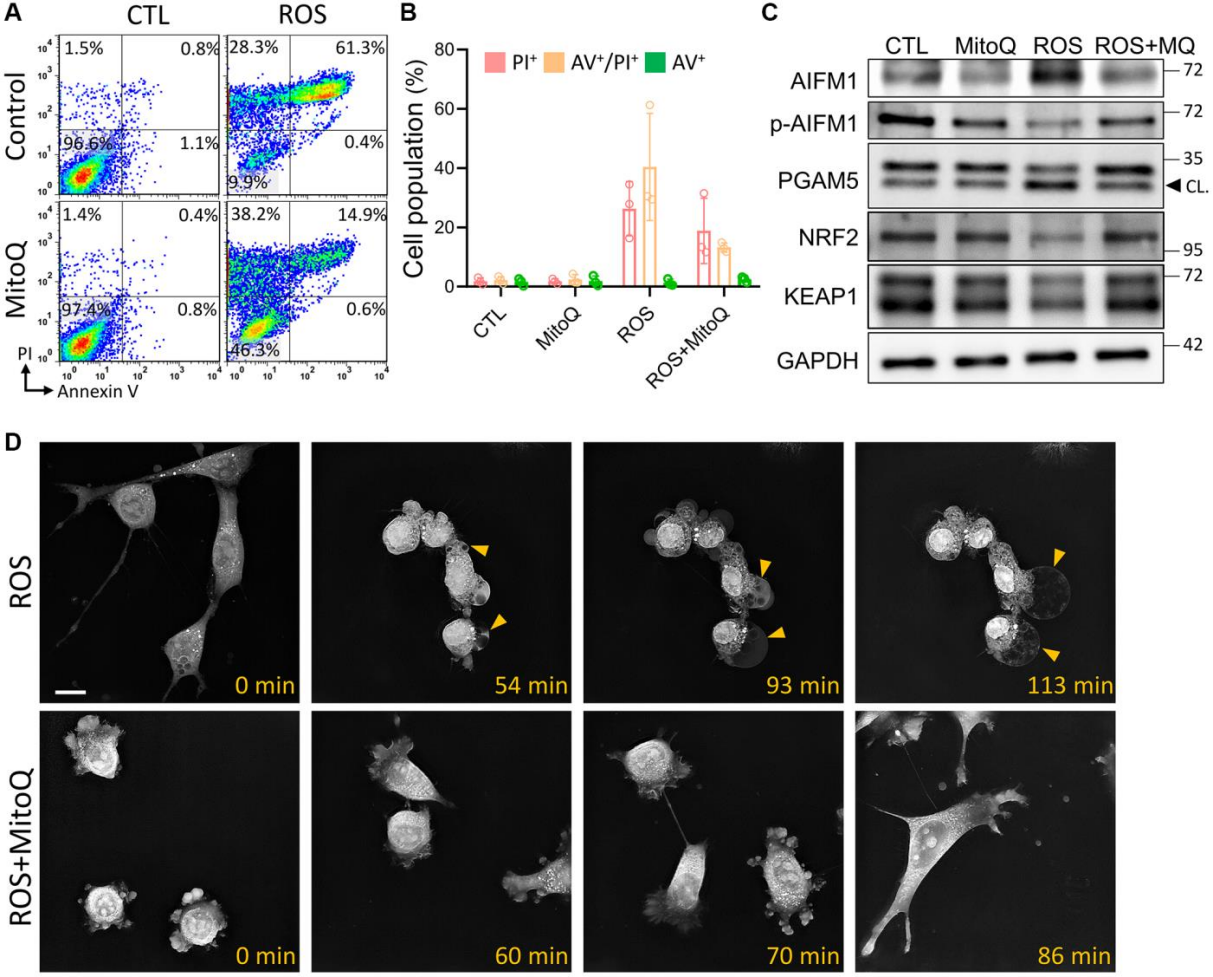
**Effect of MitoQ on ROS-induced oxeiptosis.** (**A**) Flow cytometry was performed on MitoQ treated for 24 h, followed by 0.8 mM H_2_O_2_ treatment for 4 h and double staining by annexin V/PI. (**B**) Quantification of annexin V/PI double-stained cell populations in each quadrant, respectively. (**C**) Analysis of protein levels in granule cells by immunoblotting. (**D**) The time-lapse images are used to capture pictures of cell morphology at different times. CL: cleavage form. Scale bar: 25 μm.

To verify that MitoQ protects cells from cell death caused by ROS, we performed flow cytometry 24 h after MitoQ exposure. Double staining for Annexin V/PI was used to detect phosphatidylserine out-flipping, one of the most distinctive features of cell death (Figure 3A). The results showed that MitoQ reduced HGL5 in the dead cell population (PI^+^/Annexin V^+^) by 61.3% to 14.9%. However, the cell population shifted directly to the PI^−^/Annexin V^−^ quadrant, where the cell population increased from 9.9% to 46.3% (Figure 3B). The activity of oxeiptosis-related proteins was determined using immunoblotting (Figure 3C). In addition to increasing the expression of AIFM1, MitoQ decreased the expressions of PGAM5, NRF2, and KEAP1. These findings pointed to the activation of the oxeiptotic cascade as one of the mechanisms of the ROS-induced cell death in the granulosa cells (Figure 3C). We further captured the morphological changes in MitoQ-protected granule cells exposed to ROS using time-lapse microscopy (Figure 3D and Supplementary Videos 1 and 2). In the ROS group, microbubbles began to appear around the granulosa cells at the 54th min. When exposed to ROS for a longer period of time, these microvesicles became larger, accompanied by the gradual disappearance of the cell pseudopods, and finally the cells appeared as giant vesicles. In contrast, cells exposed to ROS after the pretreatment with MitoQ remained motile and underwent mitosis. Eventually, the cells did not lose their pseudopods and appeared healthy.

### MitoQ normalizes the decrease in oxygen consumption rate under oxidative stress

**Figure 4:**
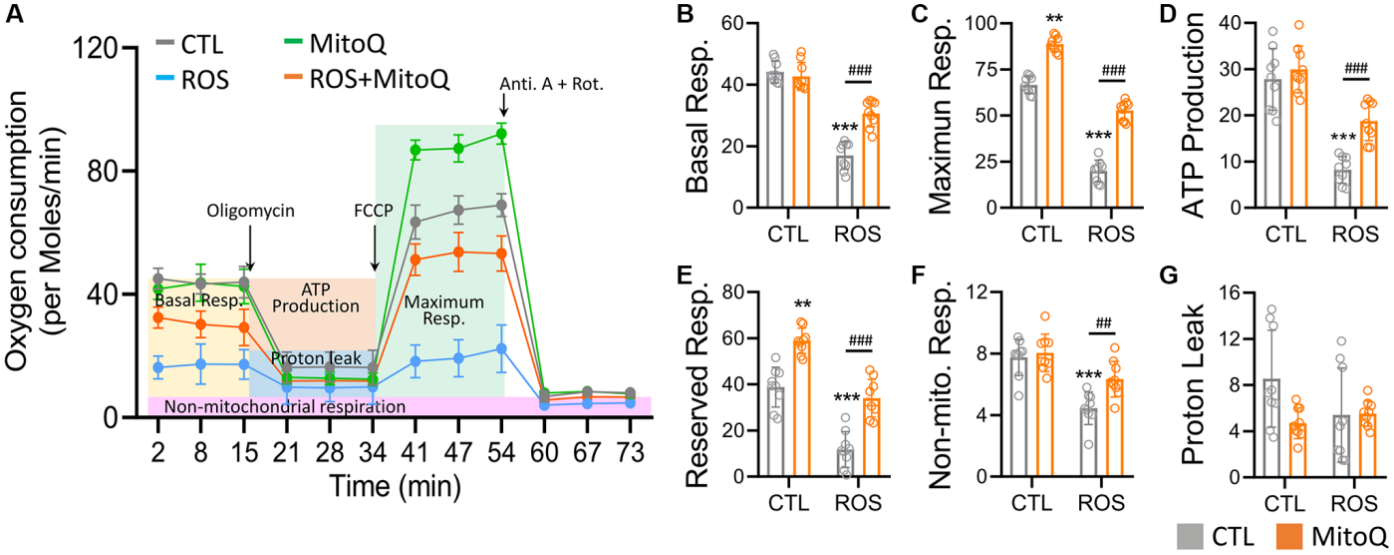
**MitoQ elevates the mitochondrial oxygen consumption of human granulosa cells.** (**A**) Analysis of cellular mitochondrial function by oxygen consumption rate (OCR) through Seahorse Bioscience Analyzer. All OCR values for different stages of basal respiration (**B**), maximal respiration (**C**), ATP production (**D**), reserved respiration (**E**), proton leakage (**F**), and non-mitochondrial respiration (**G**) were analyzed, and control, MitoQ, ROS, and ROS/MitoQ groups were analyzed. Oligomycin (1 μM), FCCP (1 μM), antimycin (0.5 μM), and rotenone (0.5 μM). ^*^p < 0.05, ^**^p < 0.01 and ^***^p < 0.001, as compared with control. ^#^*p* < 0.05, ^##^*p* < 0.01 and ^###^*p* < 0.001 compared to ROS only.

We further analyzed the oxygen consumption using a Seahorse bioenergetics analyzer and added oligomycin to evaluate the leak values after the basal oxygen consumption rate was stabilized. We then added FCCP to analyze the maximum oxygen consumption rate and finally added antimycin and rotenone. The independent experiments were replicated three times to quantify the curve in Figure 4A. Thus, the OCR curve showed that MitoQ treatment significantly enhanced the OCR curve compared to the ROS group. The MitoQ-treated cells recovered from the low oxygen consumption induced by ROS, including basal respiration (Figure 4B), maximum respiration (Figure 4C), and ATP production (Figure 4D). It should be noted that MitoQ alone and in combination with the ROS group significantly increased the intracellular reserve capacity of the ROS group (Figure 4E). In addition, non-mitochondrial respiration was significantly lower in the ROS group than in the other three groups (Figure 4F). In contrast, there was no significant difference in proton leakage among the four groups (Figure 4G). These findings suggest that MitoQ can modulate cellular mitochondria and increase the mitochondrial turnover rate to increase intracellular energy.

### MitoQ regulates reprogramming of energy metabolism under oxidative stress

**Figure 5:**
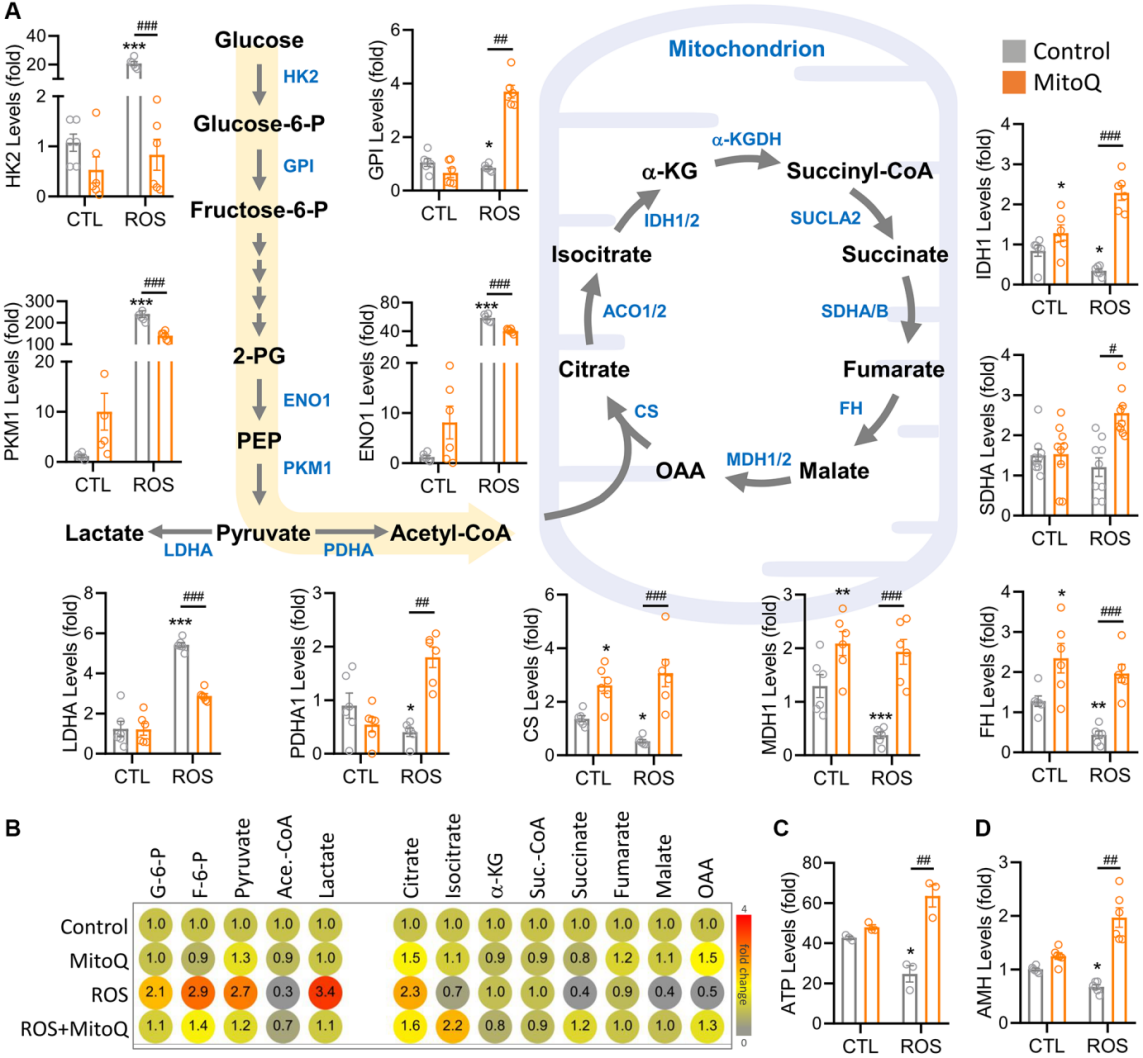
**MitoQ regulates ROS-induced reprogramming of cellular energy metabolism.** (**A**) Schematic diagram showing metabolic pathways and qPCR analysis of the levels of genes involved in glycolysis and the TCA cycle. (**B**) The heatmap showing metabolite levels of glycolysis and TCA cycle pathway by UHPLC- MS/MS analysis. (**C**) Measurements of ATP by flow cytometry. (**D**) qPCR analysis of AMH gene levels. ^*^p < 0.05, ^**^p < 0.01 and ^***^p < 0.001 compared to the control. ^#^*p* < 0.05, ^##^*p* < 0.01 and ^###^*p* < 0.001 compared to ROS only.

To determine the metabolic pathways regulated by MitoQ in human granulosa cells under oxidative stress, we examined metabolic genes and found that the levels of metabolic genes in the ROS group of cellular glycolysis and the TCA cycle were significantly restored by MitoQ (Figure 5A). The genes involved in the glycolytic pathway included HK2, GPI, ENO1, PKM1, and LDHA. Once the pyruvate-regulated acetyl coenzyme A entered the TCA cycle to produce more ATP, the genes involved were CS, IDH1, SDHA, FH, and MDH1. We further confirmed the changes in cell metabolites using an ultra-high-performance liquid chromatography (UHPLC)-MS/MS (Figure 5B). Glucose-6-phosphate, fructose-6-phosphate, and lactate levels in glycolytic genes significantly rose in the ROS group, whereas all the three enzymes significantly decreased with the MitoQ treatment. The enzymes involved in the TCA cycle, including isocitrate, succinate, malate, and oxaloacetate, were lower in the ROS group but significantly increased with the MitoQ treatment. We confirmed ATP production based on flow cytometry analysis and found that MitoQ effectively increased ATP decay caused by ROS (Figure 5C). Given the measured levels of anti-Müllerian hormone (AMH), an important biomarker of female germ cell quality, MitoQ improved the quality of human granulosa cells (Figure 5D). Overall, these data suggest that MitoQ restores reprogramming of glucose metabolism and the TCA cycle in human granulosa cells under oxidative stress.

### MitoQ enhances the meiotic maturation of aging murine oocytes

**Figure 6:**
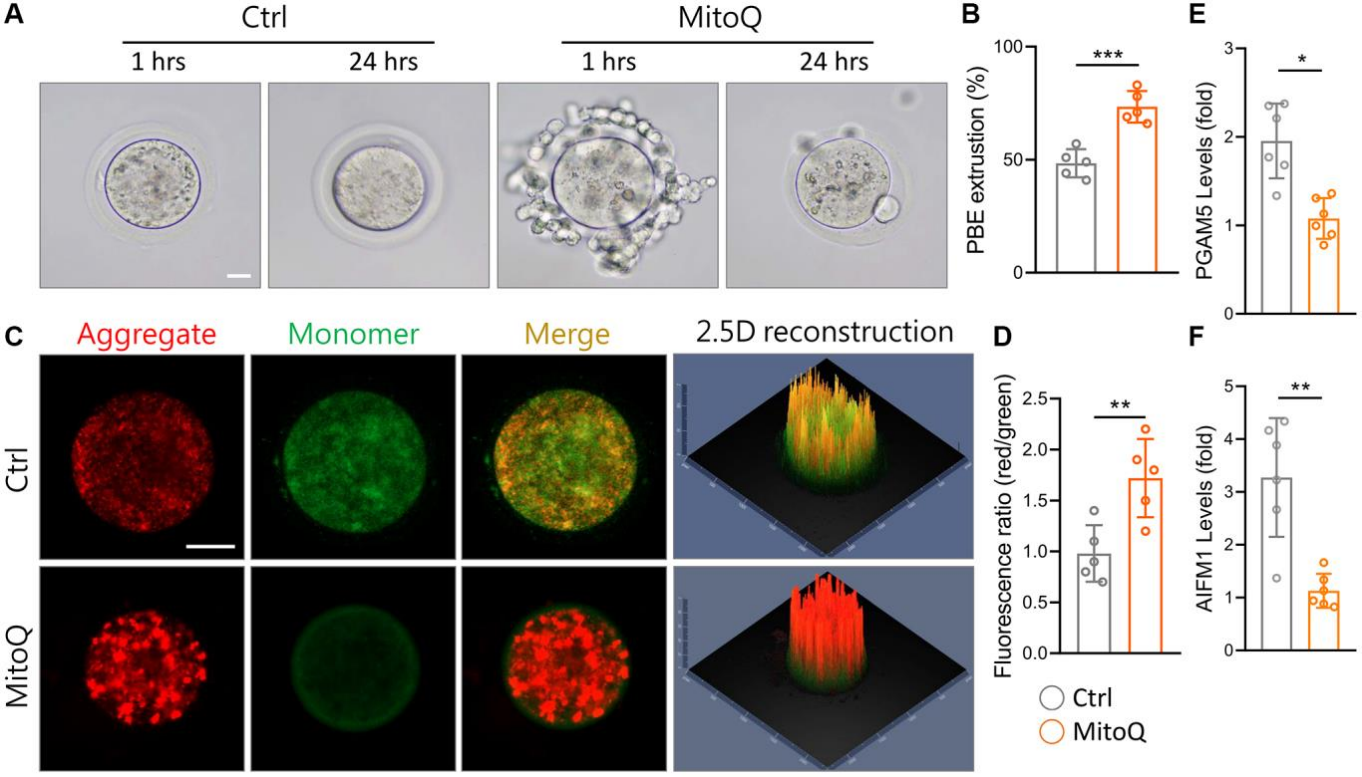
**Effect of MitoQ on *in vitro* maturation of aging mouse oocytes and mitochondrial function.** All oocytes were matured *in vitro* with 10 nM MitoQ for 24 h from 51-week-old B6 mice. (**A**) Representative images of *in vitro* maturation of oocytes from aged mice. (**B**) The quantification of first polar body extrusion from mice oocytes. (**C**) Mitochondrial membrane potential (Δψm) assessed by JC-1 staining in control and supplemented MitoQ oocytes (red, Δψm high; green, Δψm low). (**D**) Quantification of the ratio of red to green fluorescence intensity in control and MitoQ-supplemented oocytes. (**E**, **F**) The levels of oxeiptotic core genes were determined by qPCR. Scale bar, 25 μm. ^*^p < 0.05, ^**^p < 0.01, ^***^p < 0.001.

To explore the effect of MitoQ on the maturation of murine oocytes *in vitro*, we assessed oocyte maturation by observing first polar body extrusion (PBE). MitoQ was added to 51-week-old oocyte culture medium and incubated for 24 h. The percentage of mature oocytes increased with the MitoQ exposure, with a higher number of PBE (Figure 6A). The exposure to MitoQ increased PBE by 20% after 24 h of *in vitro* maturation (Figure 6B). Specifically, the oocyte maturation rate in aged mice significantly rose with the MitoQ treatment. Î” Î ¨ *Δψm* is an indicator of mitochondrial health and quality. Therefore, we evaluated *Δψm* to further determine the efficacy of MitoQ in improving poor mitochondrial quality of aging oocytes. The ratio of red/green fluorescence intensity of oocytes stained with JC-1 was used to assess the membrane potential of the cells. As was shown in Figure 6C, the green fluorescence intensity of aged oocytes was higher without the MitoQ treatment than with the 24-h treatment. The merged images showed a large amount of yellow fluorescence in the aged oocytes, whereas the MitoQ-treated aged oocytes appeared red. Reconstructed in 2.5D, the green fluorescence was higher in the untreated oocytes than in the MitoQ-treated oocytes, thus causing the yellow coloration of the oocytes. Given the ratio of red-to-green fluorescence, the MitoQ-treated group had higher membrane potential than the untreated group (Figure 6D). The levels of the oxeiptosis-related genes, including PGAM5 (Figure 6E) and AIFM1 (Figure 6F), significantly fell with the MitoQ exposure, thus resulting in the high expression of both genes. This in turn confirmed that MitoQ regulates ROS-induced apoptosis.

## DISCUSSION

Human germ cells are extremely sensitive to oxidative stress and maintain optimal oocyte quality over time [23]. Recent studies have shown that primordial oocytes escape ROS by eliminating complex I and remodeling the mitochondrial electron transport chain. Once the mitochondrial dysfunction and oxidative stress occur, a negative cycle develops in the cell. Thus, defects in mitochondrial complex I produce more free radicals [24]. The regulation of mitochondrial activity and slowing ROS production in healthy oocytes are the important issues [25, 26]. Interestingly, free radicals are normal metabolic products of cells. Under the normal physiological conditions, the production and scavenging of free radicals are dynamic [27]. Once intracellular constancy is disrupted, the accumulation of free radicals in the body can cause oxidative stress, leading to peroxidation of DNA, proteins, and lipids which in turn destroys mitochondrial complex I and eventually causes the mitochondrial dysfunction and programmed cell death [23, 28].

Coenzyme Q10 is the only naturally reproducible fat-soluble antioxidant in the body. Coenzyme Q10 can improve mitochondrial dysfunction by scavenging free radicals and reducing oxidative stress through the conversion of redox structures [29, 30]. MitoQ not only scavenges free radicals but also effectively enters the mitochondrial matrix to reduce the concentration of free radicals [31]. Coenzyme Q10 is the only naturally reproducible fat-soluble antioxidant in the human body, is also an important player in the mitochondrial electron transport chain, and is involved in the synthesis of ATP [32, 33]. Therefore, MitoQ is considered to be more effective in free radical scavenging, reducing oxidative stress, and improving mitochondrial dysfunction as well as effectively entering the mitochondrial matrix to reduce the concentration of use [34]. It also alleviates the arsenic-induced dysfunction of mitochondria-associated endoplasmic reticulum membranes (MAMs) in lung epithelial cells [35]. The above study clearly indicates that MitoQ is effective in improving cell damage and programmed cell death caused by ROS.

In this study, apoptosis was induced by a high concentration of H_2_O_2_ which induced ROS production and forced cells to initiate the oxeiptosis mechanism instead of apoptosis for a short period of time. H_2_O_2_ induced a large amount of ROS production in the cells which lowered the mitochondrial membrane potential and caused atypical cell death (Figure 3). However, the pretreatment with MitoQ improved the damage caused by ROS to mitochondria and reduced the production of free radicals in the cells (Figure 4). The cellular morphology of oxeiptosis is not well described, but time-lapse photography showed that the effect of the high ROS concentrations on the granular cells was different from those of the general apoptotic and necrotic properties. The MitoQ treatment was effective in increasing mitochondrial activity to block the germ cell damage caused by the high ROS concentrations. We speculate that oxeiptosis is a specific self-protective bailout mechanism against oxidative stress that can rapidly activate AIFM1 to induce apoptosis. Previous studies have reported that MitoQ rapidly accumulates in the mitochondria of Sertoli cells and upregulates the mitochondrial kinetic proteins Mfn2 and Drp-1. The mechanism involved the significant activation of the Keap1-Nrf2 antioxidant defense system, which effectively inhibited triptolide-induced oxidative stress damage in the testis [36]. This study may be a possible way for MitoQ to regulate KEAP1/NRF2 and further drive the cells toward apoptosis.

MitoQ protected the germ cells from the ROS damage through the three major pathways: mitochondrial-dependent, oxeiptosis, and metabolic shift for cellular protection and regulation. The antioxidant activity of MitoQ exerted at least several specific points to protect cellular mitochondria. First, MitoQ not only directly inhibited the overproduction of DCFHDA and MitoSox (Figure 1), but also improved the mitochondrial quality (Figure 4) and increased the mitochondrial length significantly (Figure 2). Second, MitoQ inhibited mitochondrial membrane potential depolarization to avoid the release of lethal proteins due to potential differences (Figure 6). The change in the mitochondrial potential difference was also involved in the energy metabolism conversion and synthesis. Finally, MitoQ directly inhibited the translocation of Drp1 to the mitochondria (Figure 2) to reduce the excessive mitochondrial division and maintain the mitochondrial morphology. These key factors, including KEAP1, PGAM5, NRF2, and AIFM1, were the multiple regulators of complex messaging. In addition, MitoQ protected the cellular energy production through metabolic reprogramming to provide sufficient energy to maintain the cellular physiological functions, thus reducing apoptosis.

## CONCLUSION

Overall, MitoQ may be directly related to mitochondrial biogenesis and has the ability to strengthen mitochondria to maintain the stem cell proliferation and differentiation. Previous studies have suggested that MitoQ regulates PGC1A. In this study, MitoQ was used to dual-regulate and protect the quality of mitochondria. The strategy of handling MitoQ deserves further evaluation and application in the future so that a better understanding is achieved about how to help cells to generate a direct or indirect defense against oxidative stress or improve their repair ability as well as how to overcome the oxidative stress generated by the *in vitro* culture environment of oocytes and limit the unfavorable factors of cell maturation *in vitro*. In this context, the provision of clinical artificial reproduction is also of great research value.

## Supplementary Materials

Supplementary Video 1

Supplementary Video 2

Supplementary Table 1
