# Supplementary material for: Mitoquinone shifts energy metabolism to reduce ROS-induced oxeiptosis in female granulosa cells and mouse oocytes
Source: Aging (Albany NY). 2023 Jan 9;15(1):246–60. doi: 10.18632/aging.204475 (PMC9876626; doi:10.18632/aging.204475)
Supplement: Supplementary Table 1 [file aging-15-204475-s003.pdf]

SUPPLEMENTARY TABLE

Supplementary Table 1. Primer sequences designed for RT-PCR.

Primers	Forward	Reverse
AIFM1	F-CGTAAAGCCGTAAAGGCATCG	R-AGAAGACGTAATCCCGCTGTT
PGAM5	F-CAA ACC CAG AAT TGT TCT CCT T	R-ATG TGG TCT TCC TGA ATC CCT
HK2	F-TCCGTAACATTCTCATCGATTTCA	R-TGTCTTGAGCCGCTCTGAGAT
GPI	F-GACCCAGCACCCCATACG	R-CAAGAAGTTGGCCAGGAGGAT
ENO1	F-TGGGAAAGCTGGCTACACTGA	R-CTCGGAGGCCGCTACGT
PKM1	F-TCTGAGCGGTCTTTGCTAGTGA	R-TGACATAATGCTCCCCTTTTGG
LDHA	F-GAAGCGGTTGCAATCTGGAT	R-GGTGAACTCCCAGCCTTTCC
PDHA1	F-ACCCCACAGACCATCTCATCA	R-CCCCGGGTGAAAGTAAAGC
PDHB	F-AACTGTGGTTTCCCATTCAAGAC	R-TTAGATAGCACTGCTGCAGCTTCT
CS-F	F-TCTGGAGCCGAGCCTTAGG	R-GACCCTCTGTGCTCATGGACTT
MDH1	F-GCTGTCATCAAGGCTCGAAAAC	R-GGTCACAGATGGCTTTTGCA
KGDH	F-TGC TCG GCA ATT CAG TCA TC	R-GCC AGT GTG CCA TCG CTT A
IDH1	F-CGG AAC CCA AAA GGT GAC AT	R-TGG CAA CAC CAC CAC CTT CT
AMH	F-CGC TGC TTC ACA CGG ATG ACC	R-GGT GGC GAC TCC TCG AGT TCC
RNU6-1	F-CTCGCTTCGGCAGCACATATACT	R-ACGCTTCACGAATTTGCGTGT C
